# Adult Idiopathic Ileocolonic Intussusception: A Case Report

**DOI:** 10.7759/cureus.20393

**Published:** 2021-12-13

**Authors:** Rodney C Sena, Garett Forosisky

**Affiliations:** 1 Emergency Medicine, Inspira Health Network/Inspira Medical Center Vineland, Vineland, USA

**Keywords:** hematochezia, abdominal pain, idiopathic intussusception, ileocolonic intussusception, adult intussusception

## Abstract

Abdominal pain is a common chief complaint seen in the emergency department (ED), both in adult and pediatric populations. In pediatric emergency medicine, one diagnosis that is often considered is intussusception; in adult emergency medicine, this is typically less common. The classic teaching in adults is that the most common cause of intussusception is malignancy. In the following case report, however, the etiology of intussusception was not from malignancy but rather idiopathic. This case report describes an elderly female presenting to the ED for abdominal pain, nausea, vomiting, and hematochezia. She underwent abdominal imaging with findings concerning for intussusception; this necessitated urgent operative intervention. She was found to have ileocolonic intussusception. Tissue biopsy was sent for analysis to evaluate for malignancy. This was negative. While emergency physicians often keep intussusception high on their list of differential diagnoses when dealing with pediatric patients with abdominal pain, it is rarely considered in the adult population. Abdominal pain is one of the most common chief complaints seen in emergency medicine. Intussusception should definitely be considered as this could be the etiology of abdominal pain and, in rare circumstances, the potential etiology for an acute abdomen.

## Introduction

Intussusception is commonly seen in the pediatric population; however, it can also manifest in adults. This case report describes an elderly female who presented to the emergency department (ED) for evaluation of several hours of abdominal pain, nausea, and bloody diarrhea. She was ultimately diagnosed with ileocolonic intussusception. Despite the classical teaching that the leading cause of adult intussusception is malignancy, this case demonstrates that idiopathic intussusception should be included in the differential diagnosis.

## Case presentation

A 71-year-old female presented to the ED for evaluation of abdominal pain, nausea, vomiting (nonbloody, nonbilious), and grossly bloody diarrhea. Symptoms started several hours prior to ED arrival and were gradually worsening. She complained of a diffuse cramping sensation in the abdomen. She reported three episodes of grossly bloody diarrhea and stated that she had no previous episodes of bloody stools. Her medical history included hypertension, diabetes mellitus, interstitial lung disease, and pulmonary hypertension. She had no relevant surgical history except for a few minor orthopedic procedures. Review of systems revealed a transient lightheadedness sensation which occurred while en route to the ED that resolved spontaneously prior to arrival. Her physical exam was benign including a soft abdomen that was nondistended. There was no abdominal tenderness. Digital rectal exam revealed hematochezia without internal or external hemorrhoids.

**Figure 1:**
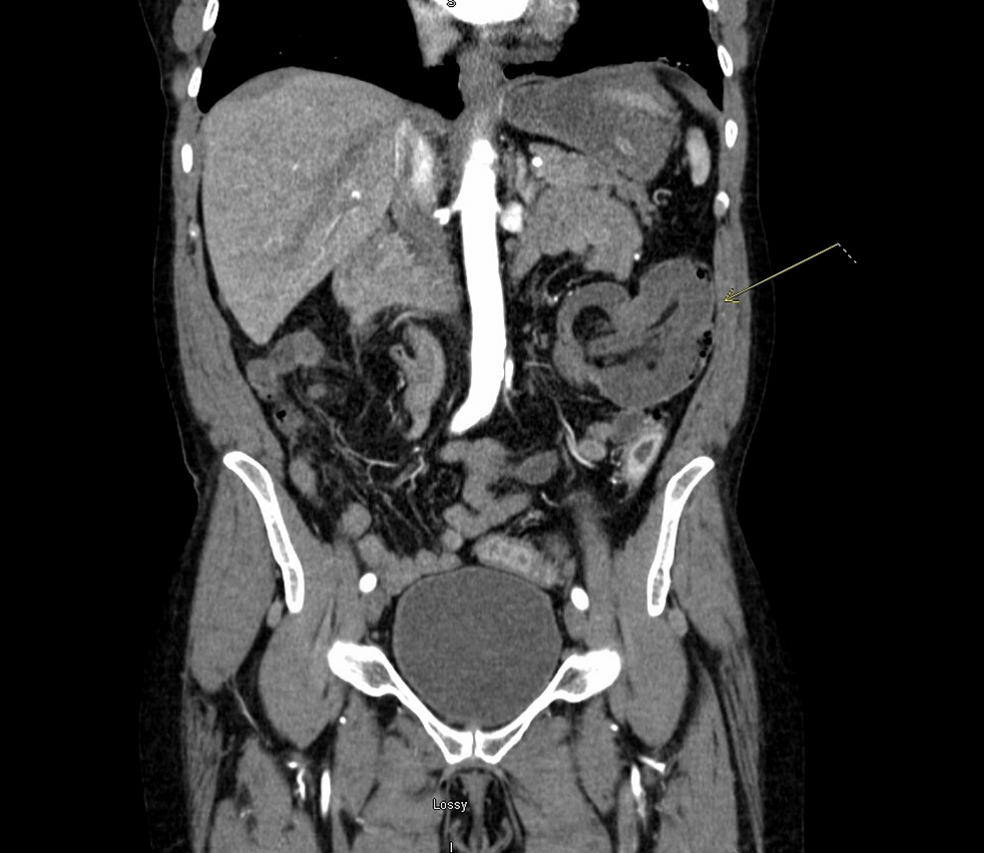
Coronal image of computerized tomography angiography of the abdomen and pelvis with concern for intussusception of the colon in the area of the splenic flexure.

Her workup included the following laboratory studies: complete blood count, metabolic panel, hepatic profile, and serum lactate. White blood cell count was mildly elevated, hemoglobin and hematocrit were stable, and serum lactate was normal. There were no acute electrolyte or hepatic panel abnormalities. Computerized tomography (CT) angiogram of the abdomen and pelvis revealed suspected colocolonic intussusception at the level of the splenic flexure with pneumatosis, pericolonic fat stranding with trace ascites (Figure 1). The radiologist did not comment on any other abnormalities of the bowel. At this point, the general surgeon on call was contacted who reviewed the images and arranged for urgent laparoscopy.

Intraoperatively, the splenic flexure of the colon (the area of concern according to preoperative imaging) was evaluated and found to be normal. The colon distal to the splenic flexure was also found to be normal. As the surgeon followed the colon proximally from the splenic flexure, the patient was found to have ileocolonic intussusception with distal bowel edema and evidence of ischemia. There were no adhesions present. She underwent right hemicolectomy with primary anastomosis. Tissue pathology revealed edematous changes with areas of focal ulceration consistent with acute and chronic inflammation. There was no evidence of adenoma or carcinoma. Patient subsequently had an uncomplicated postoperative course and was discharged home.

## Discussion

Adult intussusception is exceedingly rare, with approximately 95% of intussusception cases occurring in the pediatric population [1]. Adult cases are often associated with coexisting neoplasm which acts as a lead point, leading to the change in motility and allowing for the invagination of a segment of bowel. Approximately 60% of adult small bowel intussusception cases are caused by benign lesions, with 30% caused by malignancy and the remaining 10% idiopathic. In cases of adult colonic intussusception, approximately 60%-65% are caused by malignancy. Previously reported cases have determined intussusception to be the cause for an adult patient with an acute abdomen [2-5].

The Mayo Clinic and Mayo Medical School in Rochester, MN, conducted a retrospective study over a 13-year period from 1996 to 2008 which included 148 adult patients diagnosed with intussusception and sought to further describe these patients [6]. In their cohort, they found a mean age of 48 years with the most common presenting symptom being abdominal pain. Other presenting symptoms included nausea, vomiting, diarrhea, and bloody stools, while 20% of their population was asymptomatic. The most common location for intussusception was enteroenteric in 80%, with other locations being ileocolonic (10%), colocolonic (7%), and gastroenteric (1%). The etiology was unknown in 40% of cases, 15% attributed to malignancy, 13% due to benign neoplasm, and 10% from adhesions. A group in Japan performed a similar retrospective review and identified 44 adult patients treated for intussusception at their institution [7]. Again, the most common presenting symptom was abdominal pain followed by vomiting, diarrhea, nausea, and then melena. The etiology in this group was found to be tumor associated in 77.3% of cases, 11.4% due to adhesions, and 11.4% idiopathic. The Japanese study reported 41 of their 44 patients being treated surgically, whereas the American study reported 77 of their 148 patients treated surgically within one month.

Aside from the final diagnosis, another interesting aspect of the presented case is the discrepancy between imaging findings versus surgical findings. The radiologist did not comment on any radiographic abnormalities in the area of the ileocolonic junction. Given the pathophysiology of intussusception and the presence of continuing peristalsis, one possible explanation is a transient intussusception at separate locations. Zissin et al. reported a case of transient colocolic intussusception caused by a mass in the sigmoid colon [8]. Two separate CT scans were obtained five minutes apart; the first showed the intussusception, whereas the second did not. Additional cases are reported of transient small bowel intussusception in adults where the abnormality is identified on imaging and surgical findings are normal [9-11]. We speculate that the presented patient had idiopathic intussusceptions in two separate locations, with the first being transient in the area of the splenic flexure and the second being surgically present at the ileocolic junction.

## Conclusions

Medical students and physicians-in-training are often taught some form of the adage, “If one does not think of the diagnosis, one cannot make that diagnosis.” Abdominal pain is one of the most common presenting chief complaints for visits to the ED. It can have a broad differential diagnosis. This case report serves as a reminder for emergency physicians to include intussusception as a possible diagnosis when working through an adult patient with abdominal pain, particularly if there is evidence of hematochezia. While malignancy is certainly a common cause of intussusception, it is just as likely for cases to be idiopathic.

## References

[1] Walls RM, Hockberger RS, Gausche-Hill M (2018). Rosen’s Emergency Medicine: Concepts and Clinical Practice. Ninth Edition. https://www.us.elsevierhealth.com/rosens-emergency-medicine-concepts-and-clinical-practice-9780323354790.html.

[2] Lu T, Chng YM (2015). Adult intussusception. Perm J.

[3] Gange ER, Grieco MA, Myers SD, Guenther TM (2020). Idiopathic adult intestinal intussusception: a rare cause of an acute surgical abdomen. J Surg Case Rep.

[4] Patel S, Eagles N, Thomas P (2014). Jejunal intussusception: a rare cause of an acute abdomen in adults. BMJ Case Rep.

[5] Timothy Adewale A, Rowe SM, Solomon GM (2019). Colocolonic intussusception in an adult cystic fibrosis patient. J Cyst Fibros.

[6] Lindor RA, Bellolio MF, Sadosty AT, Earnest F 4th, Cabrera D (2012). Adult intussusception: presentation, management, and outcomes of 148 patients. J Emerg Med.

[7] Honjo H, Mike M, Kusanagi H, Kano N (2015). Adult intussusception: a retrospective review. World J Surg.

[8] Zissin R, Gayer G, Konen O, Shapiro-Feinberg M (2000). Transient colocolic intussusception. Clin Imaging.

[9] Napora TE, Henry KE, Lovett TJ, Beeson MS (2003). Transient adult jejunal intussusception. J Emerg Med.

[10] Aref H, Nawawi A, Altaf A, Aljiffry M (2015). Transient small bowel intussusception in an adult: case report with intraoperative video and literature review. BMC Surg.

[11] De Robles MS, O'Neill RS, Young CJ (2020). Transient jejuno-jejunal intussusception in an anabolic steroid user: a case report. Int J Surg Case Rep.

